# pH-Responsive Chitosan Films Enriched with NADES-Extracted Wine Lees Anthocyanins for In Situ Food Monitoring

**DOI:** 10.3390/gels11090676

**Published:** 2025-08-24

**Authors:** Panagiotis E. Athanasiou, Michaela Patila, Renia Fotiadou, Iro Giotopoulou, Nektaria-Marianthi Barkoula, Epaminondas Voutsas, Haralambos Stamatis

**Affiliations:** 1 Laboratory of Biotechnology, Department of Biological Applications and Technology, University of Ioannina, 45110 Ioannina, Greece; p.athanasiou@uoi.gr (P.E.A.); mpatila@uoi.gr (M.P.); p.fotiadou@uoc.gr (R.F.); 2 Department of Materials Science and Engineering, University of Ioannina, 45110 Ioannina, Greece; i.giotopoulou@uoi.gr (I.G.); nbarkoul@uoi.gr (N.-M.B.); 3 Laboratory of Thermodynamics and Transport Phenomena, School of Chemical Engineering, National Technical University of Athens, Zografou Campus, 15780 Athens, Greece; evoutsas@chemeng.ntua.gr

**Keywords:** winemaking by-products, anthocyanins, intelligent films, NADES, pH indicators

## Abstract

Due to the prevalence of plastic-packaged foods, as well as the need for real-time food monitoring by consumers, reducing plastic pollution is essential for a healthier environment and nutrition. For these reasons, in this work, biodegradable pH-responsive chitosan films enriched with wine lees-derived anthocyanins were produced, and their pH sensitivity was thoroughly evaluated. Optimization of ultrasound-assisted extraction using ethanol/water mixtures as conventional solvents was conducted and the optimal conditions (regarding total anthocyanin content, total phenolic content, and antioxidant activity) were used to perform a screening of extraction with 16 different Natural Deep Eutectic Solvents. Among them, choline chloride: butylene glycol (1:4), at a concentration of 50% *v*/*v* in water, demonstrated the highest anthocyanin recovery and was selected for the preparation of the films. The resulting films exhibited an excellent colorimetric response to pH changes, with a color difference (ΔE) exceeding 6.8 at all tested pH values, improved mechanical properties, nearly zero UV permeability, and their antioxidant activity increased by up to 6.1-fold compared to pure chitosan film. Finally, the film was applied in detecting the freshness of pork meat, exhibiting a ΔE of 15.3. The results demonstrate that the developed film is a promising alternative for intelligent, bioactive, and biodegradable food packaging for food applications.

## 1. Introduction

In recent years, as most food products are packaged, the best preservation of food and monitoring of its freshness are mandatory for the consumer’s experience [1]. In parallel, sustainable food packaging is of the utmost importance for a greener environment with less microplastics [2]. For these reasons, natural polymer gels, such as chitosan and gelatin gels, are used to produce non-toxic, biodegradable bioactive films for alternative food packaging applications [3,4,5]. Chitosan films, containing anthocyanins from various sources, like vinasse and barberry, can also be used as pH-responsive films due to their ability to change color as a response to pH changes. These films are used to detect the pH changes that occur during the spoilage of food, such as shrimps and meat, meeting the consumer’s need to know about the freshness and safety of the products [4,6].

Chitosan is derived from the deacetylation of chitin, a polysaccharide found in abundance in nature and mainly in the shells of crustaceans, such as shrimps, crabs, etc. [7]. The casting of chitosan gels to prepare films for food packaging applications is well described due to their high biodegradability and their antioxidant and antimicrobial properties [8]. Additionally, incorporating natural phenolic extracts from winemaking by-products (e.g., grape pomace, wine lees, grape seed) in the film matrix often enhances their antioxidant and antimicrobial properties [8,9,10]. Similarly, chitosan has also been used to produce smart pH-responsive packaging films by adding extracts rich in natural pigments. For example, barberry, aronia, and red cabbage have been successfully utilized as anthocyanin-rich sources in the fabrication of pH-sensitive chitosan-based films [6,11,12]. However, the poor mechanical properties of chitosan films limit their use without the addition of a proper plasticizer such as glycerol.

Anthocyanins, a subclass of (poly)phenolic compounds, are natural pigments responsible for the red, blue, and purple colors in plants [13]. In response to the pH variation, anthocyanins undergo structural changes, resulting in a visible color shift [14]. Depending on pH, different anthocyanin structures predominate, resulting in pink-red colors under acidic conditions and green to yellow hues under alkaline conditions [15,16]. This property makes anthocyanins a promising tool for developing films that can detect pH changes. However, due to the high cost of pure anthocyanins, a sustainable solution is the extraction of anthocyanins from natural sources and agro-industrial wastes, following the principles of zero waste management and the circular economy [13,17].

The traditional extraction methods for anthocyanins involve the use of hydroalcoholic mixtures, typically ethanol or methanol [18]. However, according to the EU environmental policy targets, there is a general need to reduce the use of organic volatile solvents because they are flammable and often toxic [19,20]. A promising alternative is the use of Natural Deep Eutectic Solvents (NADES). NADES are mainly composed of a hydrogen bond donor (e.g., an alcohol or a carbohydrate) and a hydrogen bond acceptor (e.g., a quaternary ammonium salt), forming a liquid with a lower melting point compared to their parental materials [21]. They have gained considerable interest owing to their low cost, toxicity, and volatility, as well as their easy preparation and high biodegradability [22]. Due to their characteristics and the high stability of phenolic compounds within these solvents, NADES already have an extended use in the extraction of bioactive compounds from numerous natural sources [22,23].

Wine lees, a by-product of the winemaking process that precipitates on the barrel bottom, are an excellent source of phenolic compounds, including anthocyanins [24]. Many non-conventional extraction processes, such as ultrasound-assisted extraction (UAE), microwave-assisted extraction (MAE), and extraction using NADES, have already been proposed for extracting bioactive compounds from wine-related by-products, with recovery yields superior to conventional extraction methods [23,24]. For example, various choline chloride-based NADES have been used effectively to extract anthocyanins from wine lees using UAE [25]. Similarly, choline chloride- and betaine-based NADES were used for grape pomace anthocyanins recovery with promising results [26,27].

In parallel, the applications of NADES are not limited to the extraction of bioactive compounds from natural sources. Chitosan films are often characterized by poor mechanical properties, especially low elongation [28,29]. Traditionally, common plasticizers (e.g., polyols, such as glycerol) or other additives, like proteins and lipids, are used to enhance the flexibility of these films [28]. Therefore, NADES have been used as plasticizers in natural polymer films, serving as sustainable alternatives to conventional plasticizers and resulting in improved mechanical properties [29,30,31]. This plasticizing effect is primarily attributed to the hydrogen bond network of the NADES, which increases the free space between the polymeric chains of chitosan, thereby leading to enhanced polymer mobility [32,33].

Taking into account the aforementioned considerations, this study focuses on the development of pH-responsive chitosan films, adding a rich-in-anthocyanins wine lees extract obtained using an NADES/water mixture as an extraction solvent. The extraction conditions were optimized by focusing on the anthocyanins and phenolic compounds recovery, as well as the antioxidant activity of the extracts. Under the optimized conditions, sixteen 50% (*v*/*v*) NADES/water mixtures were used to investigate which was the most efficient for wine lees anthocyanins extraction, against 50% (*v*/*v*) ethanol/water as a control solvent. The final extract was further characterized and used as an additive in chitosan films. The produced film was characterized in terms of its colorimetric response to pH variations, antioxidant capacity, and mechanical properties, and was subsequently applied for monitoring the freshness of meat. To the best of our knowledge, this is the first report on the direct incorporation of wine lees-derived anthocyanin extract, obtained using NADES, into chitosan films, simultaneously providing colorimetric pH sensitivity and plasticizing functionality.

## 2. Results and Discussion

### 2.1. Optimization of Extraction

Ethanol is one of the most commonly used solvents for the extraction of phenolic compounds from various sources. Anthocyanins, a member of the phenolic compounds family, are well soluble in ethanol/water mixtures due to their polarity and hydrophilic character [13]. In this work, ethanol was used as a typical solvent to determine the optimal extraction conditions, employing ultrasound-assisted extraction.

**Figure 1:**
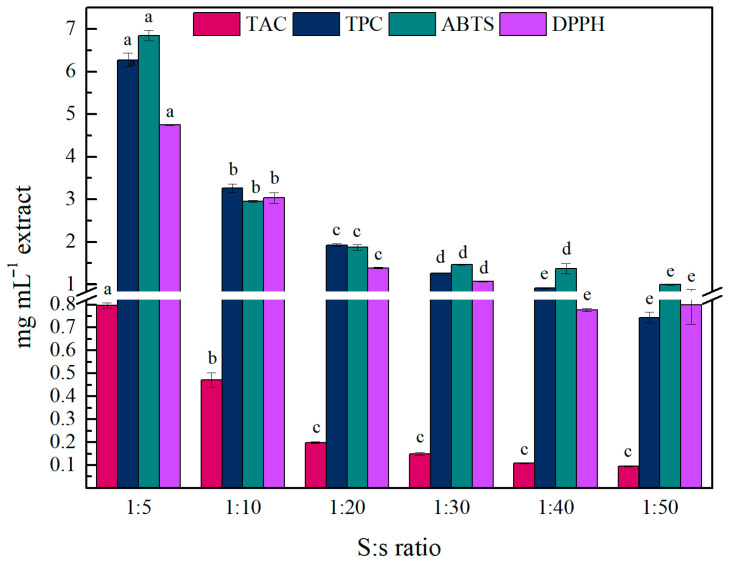
Effect of S:s ratio on the TAC, TPC, and AA of the obtained wine lees extract. Different lowercase letters indicate differences in significance (*p* ≤ 0.05) in Tukey’s multiple range test. The values for TAC, TPC, and ABTS/DPPH are expressed as mg C_3_GE mL^−1^ extract, mg GAE mL^−1^ extract, and mg TE mL^−1^ extract, respectively.

Firstly, the solid-to-solvent (S:s) ratio was investigated, and the total anthocyanin content (TAC), total phenolic content (TPC), and antioxidant activity (AA) values of the liquid extracts were determined. As shown in Figure 1, an S:s ratio of 1:5 was the most effective for recovering anthocyanins (0.80 ± 0.01 mg C_3_GE mL^−1^ extract) and phenolics (6.28 ± 0.17 mg mL^−1^ extract), resulting in the highest antioxidant activity in both DPPH (4.75 ± 0.01 mg TE mL^−1^ extract) and ABTS (6.85 ± 0.12 mg TE mL^−1^ extract) assays. As the S:s ratio decreased, all these values significantly declined, which could be mainly attributed to the phenomenon of dilution and the solvent saturation effect at higher S:s ratios [17,34]. Moreover, the proportional change observed in AA with the increase in TAC and TPC values indicates that polyphenolic compounds found in natural extracts, such as wine lees extracts, correlate with the high bioactivity of these extracts [24,25,26]. Hence, the 1:5 ratio was used for further optimization steps as the most effective condition, maintaining the lowest solvent requirement.

**Figure 2:**
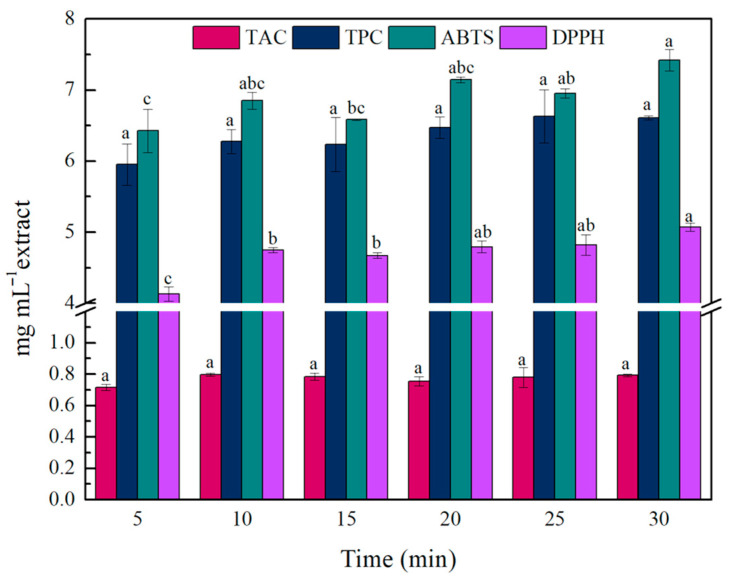
Effect of extraction time on the TAC, TPC, and AA of the obtained wine lees extract. Different lowercase letters indicate differences in significance (*p* ≤ 0.05) in Tukey’s multiple range test. The values for TAC, TPC, and ABTS/DPPH are expressed as mg C_3_GE mL^−1^ extract, mg GAE mL^−1^ extract, and mg TE mL^−1^ extract, respectively.

In the next step, the time of the UAE was investigated. As shown in Figure 2, extraction time did not significantly affect the recovery of anthocyanins. Similar trends were observed for the TPC, as no significant difference was observed between the extraction time of 5 min (5.95 ± 0.29 mg mL^−1^ extract) and the higher time intervals tested. Regarding the AA, some variation between the different extraction time intervals was better observed. Firstly, the DPPH assay revealed that increasing the extraction time led to higher antioxidant activity (up to 5.07 ± 0.01 mg TE mL^−1^ extract), while the main difference was observed between 5 and 10 min of extraction. The ABTS results also suggest that longer extraction times are associated with higher antioxidant activity. However, 5 min of UAE was as effective as 10–20 min, demonstrating that short extraction is highly efficient. Since the main objective was to maximize TAC while minimizing energy consumption, 5 min was selected as the optimal time, with satisfactory TPC and AA values. The effectiveness of low extraction time intervals during UAE, against conventional methods, for (poly)phenolic compounds’ recovery has been reported in several works [5,35,36,37]. Our results are in line with those reported by Romero-Díez et al., where UAE minimized the extraction time of wine lees’ phenolic compounds to only 5 min [37]. Similar results are also stated in another study, where 10 min of UAE of olive pomace and wine lees yielded the highest TPC values [38]. The rapid extraction could be attributed not only to the UAE but also to the starting material; it has been reported that the soluble phenolic compounds in wine lees are located on the outer space of the wine lees particles, making their extraction easier [39].

**Figure 3:**
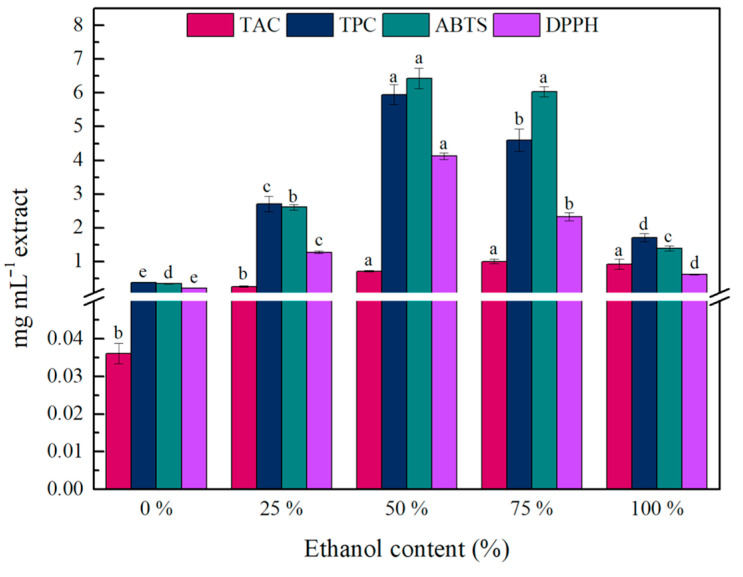
Effect of ethanol content on the TAC, TPC, and AA of the obtained wine lees extract. Different lowercase letters indicate differences in significance (*p* ≤ 0.05) in Tukey’s multiple range test. The values for TAC, TPC, and ABTS/DPPH are expressed as mg C_3_GE mL^−1^ extract, mg GAE mL^−1^ extract, and mg TE mL^−1^ extract, respectively.

Following, the ethanol content was evaluated as the final optimization factor. As can be observed in Figure 3, ethanol concentrations of 50% *v*/*v* or higher were the most effective in anthocyanins recovery, providing significantly higher yields than pure water or 25% ethanol. However, non-significant changes were observed between 50–75–100% *v*/*v* ethanol. So, 50% *v/v* ethanol (yielding 0.72 ± 0.02 mg C_3_GE mL^−1^ extract) was considered the most efficient solvent for anthocyanins recovery. A similar trend also appeared in TPC values, where 50% ethanol yielded the highest TPC value (5.95 ± 0.29 mg GAE mL^−1^ extract). Furthermore, the extract obtained with 50% *v*/*v* ethanol exhibited significantly higher AA, as determined by both ABTS and DPPH antioxidant assays (6.43 ± 0.31 and 4.13 ± 0.10 mg TE mL^−1^ extract, respectively). The effectiveness of ethanol/water mixtures for phenolic compounds recovery is mainly attributed to their polarity. The polarity of these mixtures closely matches that of anthocyanins and other phenolic compounds, leading to higher extraction yields [40,41]. Moreover, according to other studies, the use of 50% *v*/*v* ethanol as a solvent resulted in the highest TPC values in the extraction of winemaking by-products [39,41,42]. Our results are in line with those reported by Tagkouli et al., where 50% *v*/*v* ethanol content was the most efficient extraction solvent for total phenolics recovery from wine lees, leading to almost 5-fold higher extraction yield than pure water [42]. Consequently, a S:s ratio of 1:5, an extraction time of 5 min, and an ethanol content of 50% *v*/*v* were identified as the optimal parameters for anthocyanins extraction.

### 2.2. Extraction Using NADES

**Figure 4:**
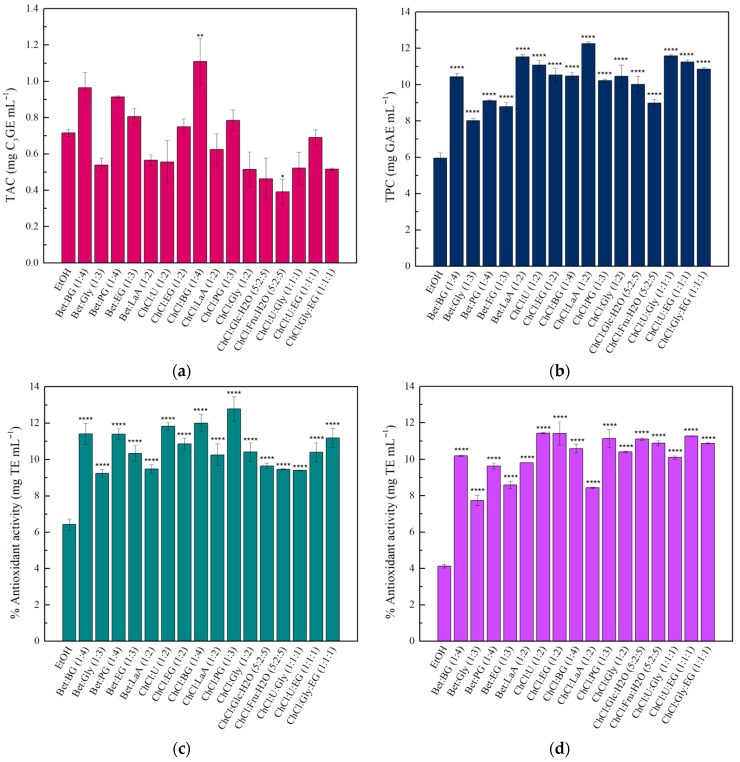
Effects of different NADES as extraction solvents on the (**a**) TAC, (**b**) TPC, and AA using (**c**) ABTS and (**d**) DPPH assays, of the obtained wine lees extract. Asterisks indicate significant differences (* (*p* < 0.0332), ** (*p* < 0.0021), and **** (*p* < 0.0001)) compared to the control sample, 50% ethanol (EtOH), in Dunnett’s post hoc test. The values for TAC, TPC, and ABTS/DPPH are expressed as mg C_3_GE mL^−1^ extract, mg GAE mL^−1^ extract, and mg TE mL^−1^ extract, respectively.

While ethanol and ethanol/water mixtures are generally recognized as safe solvents, NADES are constantly gaining ground in their use as extraction solvents. For this reason, 16 different NADES were applied as alternative solvents to ethanol in the extraction process. Figure 4 summarizes the TAC, TPC, and AA values of the obtained extracts. The results showed that most of the NADES used in a 50% *v*/*v* concentration (in water) could effectively replace a 50% aqueous ethanol mixture as a solvent to recover anthocyanins from wine lees, while 14 of them exhibited TAC values similar to those obtained with ethanol as the organic solvent. Moreover, the TPC and AA activity values were higher compared to those obtained when 50% ethanol/water mixture was used as the extraction solvent. Finally, 50% *v*/*v* ChCl:BG/water was selected for further experiments, as it was the only solvent used that led to statistically significantly higher results compared to 50% *v*/*v* ethanol. This extract exhibited a TAC value of 1.11 ± 0.13 mg C_3_GE mL^−1^ and a TPC value of 10.47 ± 0.20 mg GAE mL^−1^. The antioxidant activity, as determined by both ABTS and DPPH assays, was 12.00 ± 0.48 mg TE mL^−1^ and 10.58 ± 0.24 mg TE mL^−1^, respectively.

Numerous works indicate the advantages of using NADES for phenolic compounds’ recovery from various sources. For example, Zannou and Koca used different NADES for the extraction of anthocyanins from blackberry. Similar to our results, they indicated that most of the NADES used, especially the choline chloride-alcohol NADES, were more effective for anthocyanins, flavonoids, and total phenolics recovery, compared to conventional solvents [18]. High extraction yields using NADES were also reported for grape pomace and wine lees [25,26,27]. Moreover, Bosiljkov et al. reported that the water addition (25–50% *v*/*v*) in NADES promotes the extraction of anthocyanins from wine lees, probably due to polarity changes closer to that of anthocyanins [25]. The presence of water is also crucial; it lowers the viscosity of NADES and facilitates mass transfer phenomena, leading to higher extraction yields [43,44]. The high extraction yields observed with NADES could also be attributed to their hydrophilic character and the interaction between phenolic constituents and their hydrogen bond network within these solvents, facilitating their extraction [45].

### 2.3. Appearance and UV–Vis Characterization of the Extract at Different pH Values

**Figure 5:**
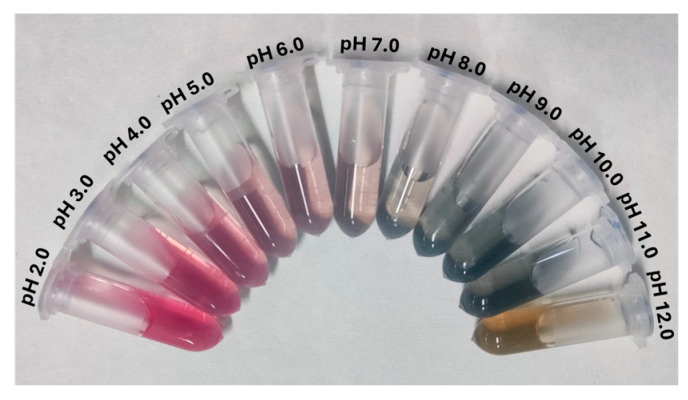
Color response of the extract at pH values 2–12 (left to right).

Anthocyanins are widely recognized as natural pH indicators due to their distinct color changes across varying pH values. As mentioned above, this property is attributed to differences in the chemical structure of anthocyanins during pH changes [15,16]. The color-changing effect of the wine lees extract in the pH range of 2–12 is shown in Figure 5. As seen, as the pH value increased, the color changed from bright pink at pH 2–3, to light pink (pH 4–5), pink-purple (pH 6–7), grey-purple (pH 8), blue-grey (pH 9–11), and yellow-brown (pH 12). Similar color variations were reported for anthocyanins extracted from grape skins [46].

**Figure 6:**
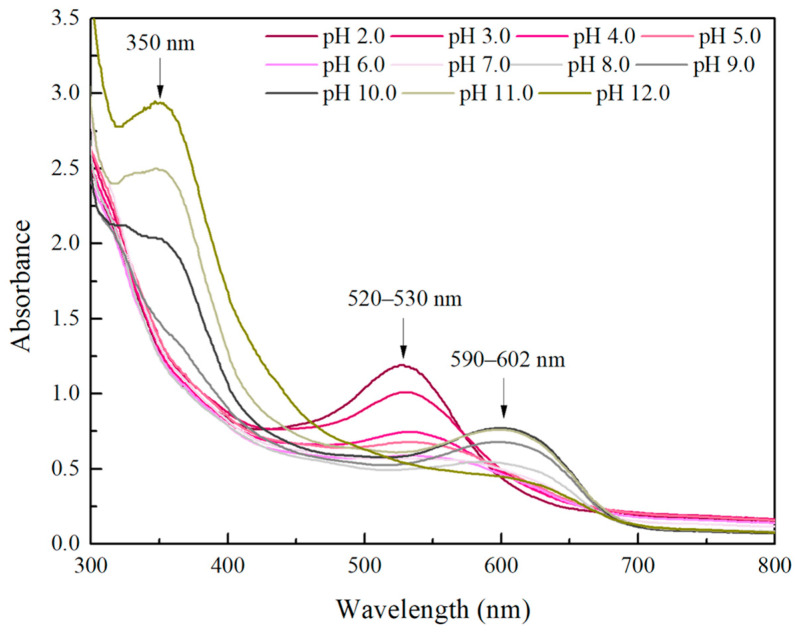
UV–Vis spectra of the extract at pH values 2–12.

**Figure 7:**
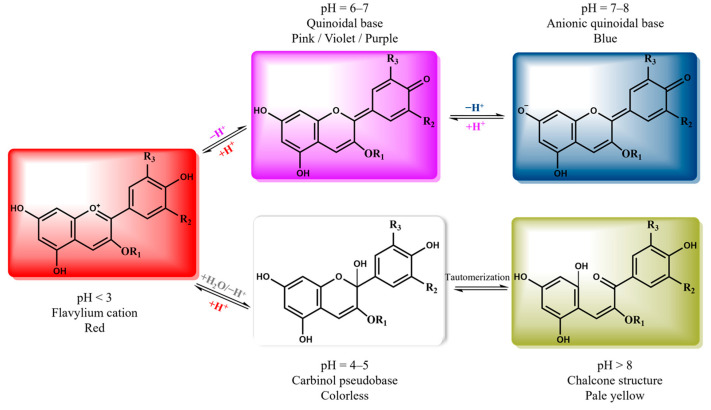
Proposed mechanism and structures of anthocyanins at different pH values (modified from [49,50,51]).

The UV–Vis spectra of the samples in the pH range were also recorded (Figure 6). At lower pH values (pH 2–6), the maximum absorption peaks were located in the range of 520–530 nm, and the intensity decreased with the pH increase. When the pH reached a value of 7, the characteristic peak faded, and at a pH value of 8, the absorption maximum was shifted to ~590 nm. Additionally, at pH values 9–11, the absorbance maximum was located at 595–602 nm, where the intensity increased proportionally with the increase in the pH value. Moreover, the intensity of the peak detected at 350 nm also increased in the pH range of 9–12. Finally, at pH 12, the intensity in the visual range decreased, and the absorbance at 350 nm displayed the highest value. Our results are in line with the literature, where alterations in the UV–Vis spectra of anthocyanin extracts in response to different pH values were described [46,47,48]. The maximum absorbance of grape skin extracts at pH values 1–5 was observed at 528 nm, while the peak was shifted to 618 nm in alkaline conditions [48]. Similar observations are reported by Kit and Kahyaoglu for grape skins; when pH < 5 or 7 < pH < 10, the maxima were found at 520 nm and 600 nm, respectively [46]. These observations are attributed to the fact that in pH values < 3, the flavylium cation is dominant, giving the red color, while when the pH increases, a quinoidal base and a pseudo base occur. Ring opening of the pseudo base or deprotonation of the quinoidal base can lead to yellowish or bluish colors, respectively. When the pH exceeds 7, anthocyanins undergo partial degradation, which decreases the concentration of the colored forms and consequently diminishes their functional properties [49]. Figure 7 represents a generalized proposed mechanism that describes the structure of anthocyanins at different pH values (modified from [49,50,51]). However, it should be noted that different anthocyanins can vary in the number and the position of hydroxyl groups, the degree of methylation, the nature of the attached sugars, and the number of aromatic or aliphatic groups attached to the sugar, resulting in variations in chemical structure and color stability [52].

### 2.4. Characterization of the pH-Responsive Films

#### 2.4.1. Appearance, Thickness, and Transparency

**Figure 8:**
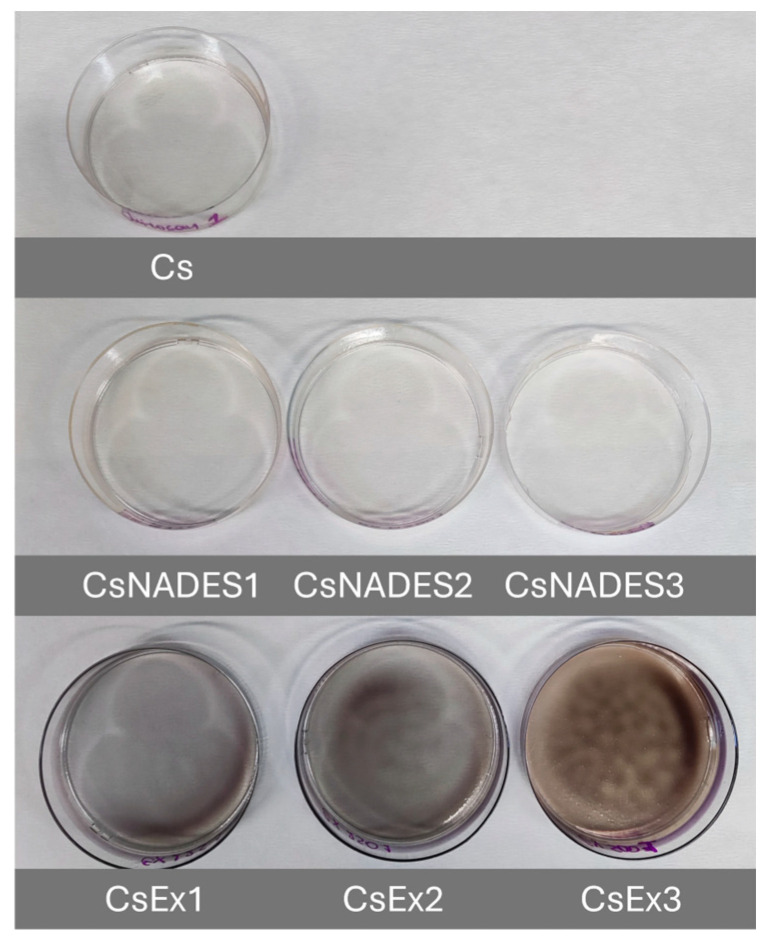
The different produced chitosan films.

The extract obtained using 50% *v*/*v* ChCl:BG (1:4) as solvent exhibited the highest TAC value; thus, it was selected to prepare the pH-responsive chitosan films. Table 1 lists the different films that were cast. As seen in Figure 8, no visible differences can be observed between the Cs and CsNADES films. The main color difference was observed in the films containing the wine lees extract; as the extract concentration increased, the color of the films progressively became more dark-blue. Moreover, the film with the highest extract loading was non-homogeneous and brownish, possibly due to partial oxidation of the phenolic constituents. To address these challenges, strategies such as improved homogenization of the chitosan-extract solution, pre-casting filtration, controlled drying, or extract encapsulation may be effective.

The films’ appearance and optical differences were also investigated by determining their thickness and opacity (Table 2). As observed, the thickness of the films increased in response to ChCl:BG and extract additions. However, no significant difference in the film’s thickness was observed between the films containing only the ChCl:BG and extract in the same proportion. Moreover, only the CsNADES3 had a statistically significantly higher thickness than the control Cs film. The slight increase in film thickness with the addition of ΝADES has also been reported by Pontillo et al., correlating this to the presence of choline chloride or other NADES components between the chitosan chains, and the formation of hydrogen bonds and electrostatic interactions, leading to swelling of the final form [32]. The same effect was also reported in another study, where the thickness of the films with low amounts of ChCl:LA did not significantly change. However, in the presence of high NADES amounts, the film’s thickness was increased [53].

Regarding the film’s opacity, the addition of NADES did not affect the opacity of the films. On the other hand, the incorporation of the extract led to a dose-dependent increase in film opacity, indicating lower transparency. According to Tukey’s test, all the films containing the extract appeared less transparent than the control films. Especially, CsEx3 (the film containing the highest extract concentration) was characterized by statistically significantly higher opacity value than CsEx1 and CsEx2. Higher opacity values of films may constitute a limiting factor for food packaging applications, due to reduced visual contact with the food, leading to poor consumer acceptability [54]. These observations are in line with another study, where the ChCl-based NADES does not affect the optical properties of chitosan films, whereas the incorporation of curcumin increases their opacity [53]. Moreover, it is known that the presence of different extracts affects the transparency of films mainly due to the presence of pigments and the alterations in the pores of the matrix, leading to lower transparency (higher opacity) [55].

#### 2.4.2. Transmittance and UV-Blocking Activity

**Figure 9:**
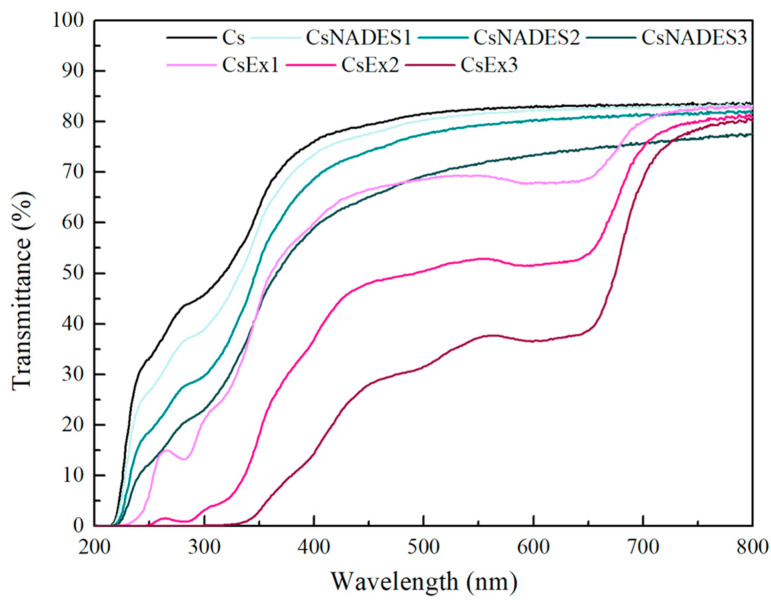
Transmittance of the different produced chitosan films.

The light transmittance of the films was recorded in the range of 200–800 nm (Figure 9). The CsEx films showed dose-dependent lower transmittance values than the CsNADES films compared to Cs. Moreover, the proportionally lower transmittance values of the CsEx films in the UV region (up to 0% transmittance in CsEx3) emphasize their ability to act as a barrier to UV radiation. This could be attributed to the phenolic constituents of the extract absorbing UV light [5]. Similar results were also reported for chitosan/polyvinyl alcohol films with *Aronia melanocarpa* anthocyanin extract [56]. Additionally, Gaviria et al. also reported that the incorporation of grape anthocyanin extract into PVA films led to zero transmittance in the range of 200–320 nm, indicating their UV-blocking capacity [57].

#### 2.4.3. Mechanical Properties of the Films

The mechanical performance of films is crucial to their application across various packaging disciplines. Thus, the mechanical properties of pristine chitosan films were assessed and juxtaposed with those of chitosan and NADES-based films with and without the extract. Following the established literature, chitosan films demonstrated brittle behavior, characterized by high Young’s modulus, and ultimate strength along with a limited strain at break (*ε*_b_). It has been previously documented that the incorporation of NADES into films decreases tensile stiffness and strength, and increases their deformability [53,58].

**Figure 10:**
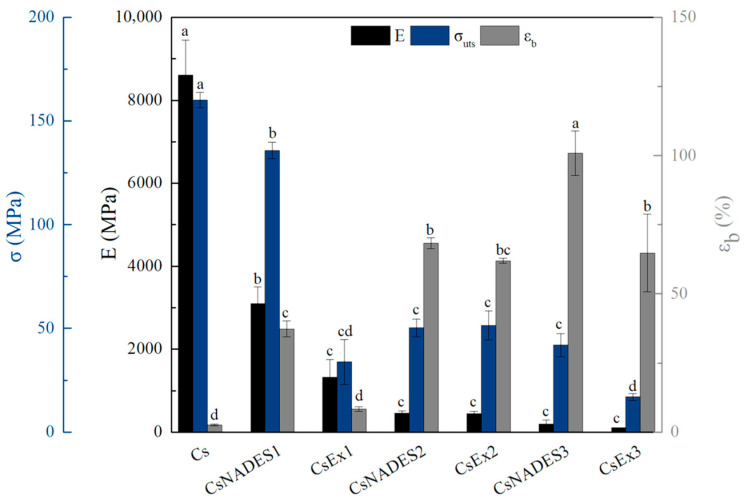
Mechanical properties of the different produced chitosan films. Different lowercase letters indicate differences in significance (*p* ≤ 0.05) in Tukey’s multiple range test.

The mechanical properties of the chitosan films are presented in Figure 10. The film with the lowest NADES concentration (CsNADES1) exhibited a reduction in Young’s modulus of about 64% and ultimate tensile strength of about 15%, along with a significant increase in strain at break of approximately 1354%. Further increase of NADES within the chitosan films gradually reduced Young’s modulus and ultimate strength while profoundly enhancing the strain at break up to approximately 3825%. These results could be attributed to the inherent nature of NADES as a plasticizing agent. The plasticizer intervenes between the molecular chains, hindering their interactions and leading to reduced stress transfer. Moreover, these chain disruptions increase free volume, thereby enhancing molecular mobility, which ultimately improves the material’s elongation at break [29,30,53,59]. Similar observations were also reported by Smirnov et al., where the addition of ChCl:LaA led to a reduction in Young’s modulus and ultimate tensile strength of about 97.5% and 48.4%, respectively, and the strain at break increased by 1614.3% at the lower NADES concentration [60]. The authors also stated that increasing the concentration of NADES resulted in a dose-dependent decrease in E and σ_uts_ values, accompanied by an increase in elongation at break [60].

The addition of the extract in the NADES-based chitosan films resulted in a non-statistically significant decrease in Young’s modulus and ultimate strength. However, films with the lowest and highest extract concentration (CsEx1 and CsEx3) exhibited a statistically significant reduction in strain at break with respect to the corresponding chitosan film with the same NADES concentration (CsNADES1 and CsNADES3, respectively). Both CsEx1 and CsEx3 also demonstrated lower Young’s modulus and ultimate strength compared to the control films. The reduction in deformability following the incorporation of extracts into polymer matrices has been documented in the established literature and is attributed to the incompatibility between the polymer and the extract [11,61,62]. Especially, the interactions between the phenolic constituents of the extract and chitosan can lead to changes in the macromolecular network of chitosan, leading to heterogeneous film structure and lower deformability [61,63,64]. Interestingly, NADES-based chitosan film with a moderate extract concentration (CsEx2) does not exhibit any statistically significant alterations in mechanical properties compared to the reference film (CsNADES2). It appears that insufficient extract concentration (CsEx1) may lead to uneven distribution within the film, creating regions with and without extract that generate localized stress points and weaken its mechanical properties. Conversely, excessively high extract concentrations (CsEx3) can disrupt the film’s structure and ultimately reduce its mechanical properties, as corroborated by other authors [62]. However, the film with an optimum, moderate extract concentration (CsEx2) mitigates both issues, maintaining a balance in the film’s mechanical properties. Overall, it is plausible to tailor the mechanical properties of the films by adjusting the concentrations of both the plasticizer and the extract in the final film.

#### 2.4.4. Water-Related Properties of the Films

The water swelling (WSw) and the water solubility (WS) of the films are presented in Table 3. The addition of ChCl:BG or extract induced a pronounced, dose-dependent decrease in the WSw of the films. The results are attributed to the presence of ChCl:BG, as no significant alterations were observed between the same loadings of ChCl:BG and the extract. The lower WSw of the films in the presence of NADES was also mentioned by other authors [3,65]. This effect could be attributed to the formation of hydrogen bonds between the ΝADES components and chitosan, reducing the interactions between chitosan hydrophilic groups and water molecules, leading to lower water intake [65]. Moreover, the solubility of the films tended to decrease at lower concentrations of ChCl:BG (or extract), but an increase was observed in higher concentrations. The same interactions of NADES and Cs described above may be responsible for this effect; at lower NADES concentrations, the hydrogen bond interactions between them and with chitosan could possibly lead to a more continuous and stable polymer structure [66], while higher concentrations of NADES may lead to increased free space and partial disruption of chitosan polymer chains [32]. Similar water solubility behavior between chitosan/PVA films containing different concentrations of choline chloride:oxalic acid DES was also reported by Yu et al. [67]. Moreover, it seems that the extract did not significantly alter the WSw and WS properties of the films to a greater extent than the ChCl:BG. Therefore, ChCl:BG is the main factor that influences the water sensitivity of the films. This is in agreement with the literature, where extracts obtained from grapes, blueberries, and parsley did not significantly affect the WS values of chitosan films [68]. Similarly, the addition of grape pomace extract did not affect the chitosan film’s WS value, confirming our observations [10].

Although lower WSw and WS values are desirable for food packaging films, the presence of swelling is necessary for the interactions of the film components with the surrounding environment, promoting the pH-sensing ability of the films [69]. On the other hand, higher WSw and WS could result in poor barrier properties and low water resistance of the films, disrupting the film’s cohesion, and making them undesirable for food packaging applications [64,65,70]. For these reasons, films like CsEx2, which demonstrate intermediate swelling behavior and controlled water solubility, may represent the optimal candidates for pH-sensing applications.

#### 2.4.5. Antioxidant Activity of the Films

The antioxidant activity of the films was evaluated through ABTS and DPPH assays, and the results are presented in Table 4. The films containing the extract exhibited higher antioxidant values, considering both assays. The effect was also dose-dependent. These results were expected, considering the antioxidant activity of the extract. As already proved and discussed, wine lees extracts are rich in phenolic compounds, the key compounds that provide them with high antioxidant activity. Moreover, the antioxidant activity of chitosan films containing wine lees extract was also proved in our previous work [9]. Lastly, the antioxidant activity using the ABTS protocol was higher than that obtained with the DPPH assay. This could be explained by differences in the mechanism and selectivity between the assays [71]. Chitosan films and phenolic compounds, due to their hydrophilic character, probably interact more easily with the ABTS^+•^ presented in the aqueous environment than the DPPH^•^ dissolved in methanol [72]. Furthermore, according to Yi et al. the release of phenolic compounds from the chitosan matrix is faster and more pronounced in aqueous media compared to organic solvents (95% ethanol) [73], probably leading to enhanced antioxidant activity of the films in the ABTS assay. Finally, steric hindrance effects are more pronounced when DPPH free radical is used, leading to reduced antioxidant activity, and thus influencing the antioxidant reaction kinetics [74].

On the other hand, films containing only ChCl:BG seemed to have a slight decrease in their antioxidant capacity, especially using the ABTS^+•^ free radical. Similar observations were reported for the effect of ChCl:Glycerol, or other NADES, on the antioxidant activity of chitosan films. The authors proposed that the addition of ChCl:Glycerol as a plasticizer decreases the antioxidant activity of chitosan films using the DPPH protocol. Moreover, they also stated differences using the different antioxidant protocols [30]. This effect may be observed due to the formation of hydrogen bonds and interactions between the NADES components and the hydroxyl and amino groups of chitosan, making them unavailable as electron donors to the free radicals, reducing their antioxidant activity.

#### 2.4.6. pH Response of the Films

**Figure 11:**
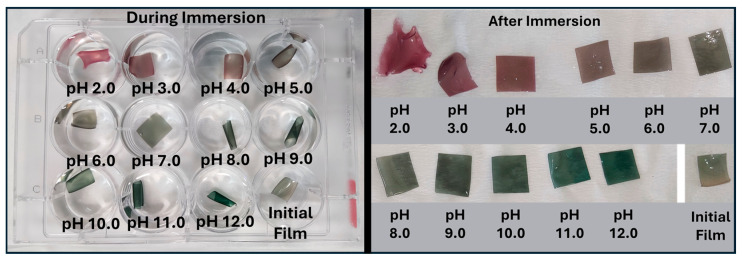
Color response of the CsEx2 film during and after the immersion in buffer with pH values 2–12.

The CsEx2 film was selected as the film with the best color appearance, a median transparency, an excellent UV-blocking ability, and balanced mechanical properties. Square pieces of this film were immersed in buffer solutions in the pH range of 2–12, and their appearance during and after immersion is depicted in Figure 11. At low pH values (2–4), the color of the film changed to reddish-pink, while as the pH rose, the color of the film turned to grey-pink (pH 5–6) and grey-green tones (pH 7–8). Finally, at pH values of 9–12, the color of the film turned to green. These observations are also shown in Table 5, where the L, a, b, and ΔΕ values are presented. As shown, all ΔE values exceeded 6.8, confirming that the films exhibited a strong color response across different pH levels. This is considered highly effective, as ΔE values above 3.5 (especially ΔE > 5.0) indicate noticeable color changes [11,75,76]. These color differences are in the same trend as PVA/gelatin films containing vinasse extract (solid winery by-product) [4] or chitosan/PVA films containing anthocyanins from red cabbage [12].

### 2.5. Monitoring of Meat Freshness

**Figure 12:**
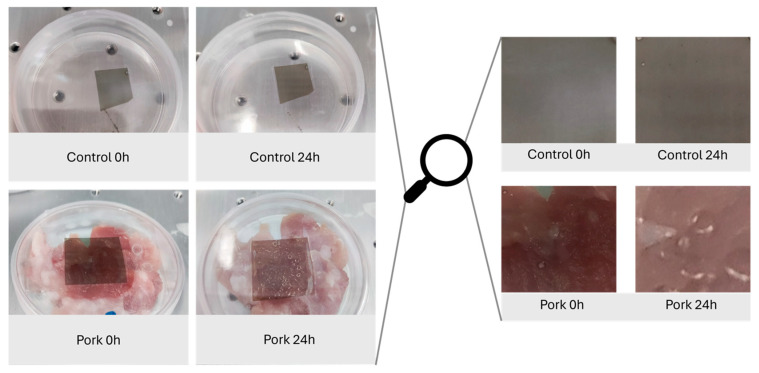
Color response of the CsEx2 film during monitoring the pork freshness for 24 h.

The color change of the film, in a closed container, after 24 h of incubation at 30 °C with (pork 24 h) or without pork (control 24 h) was evaluated. As seen in Figure 12, a non-significant color difference was observed in the control sample after 24 h of incubation. The ΔE value of the film was estimated at 2.2 ± 0.4, indicating non-observable differences and demonstrating the color stability of the film for this period. On the other hand, the ΔE value of the film after interacting with the pork for 24 h was evaluated at 15.2 ± 2.4. This value (ΔE > 5.0) indicates the clear optical difference between the film before (pork 0 h) and after (pork 24 h) the interaction. The initial color of the film resembled the color after immersing in pH 7.0 (Figure 10 and Figure 11), while after the 24 h pork interaction, the films resembled the color at pH 5.0–6.0. This suggests a decrease in the pH value, possibly due to the production of volatile organic acids during the first 24 h, detected by the film. Several volatile fatty acids (e.g., butanoic acid and acetic acid) are mainly found in spoiled meat due to microbial growth and metabolism [77]. According to another study, acetic acid was detected during the initial stages of pork spoilage, and its concentration increased during storage time [78]. Moreover, Lin et al. reported the pH values of pork meat after incubation at 4 and 25 °C. The authors observed that the pH decreases from ~6.5 to ~6.0 after the first 24 h of incubation at 25 °C (or 4 days at 4 °C) accompanied, however, by an increase in the total volatile base nitrogen (TVB-N value > 15 mg/100 g), demonstrating the spoilage of the meat [79]. Moreover, according to another study, the pH value of pork during cold storage decreased from 6.7 on day 0 to 5.89 on day 3 and remained below 6.32 for 10 days. Finally, even since the 3rd day of storage at 2 °C, the bacterial growth was sufficient to consider the meat spoiled [80].

## 3. Conclusions

Summarizing the results of this work, we demonstrated the preparation of pH-responsive chitosan films that combine plasticizing properties and pH sensitivity using a natural extract obtained with NADES. Among the different tested NADES for anthocyanins extraction, ChCl:BG was the most effective. The obtained NADES-wine lees extract was used as an additive in chitosan films, enhancing their antioxidant activity and UV-barrier effect, and providing excellent mechanical properties. The pH-sensing ability of the functional film was tested, demonstrating distinct color changes across the tested pH values. Moreover, it is notable that the L, a, b, and ΔE values were recorded using a smartphone application, highlighting the potential for practical, user-friendly commercial deployment. Finally, the film was used to monitor pork freshness, showing a clear color shift during the initial stages of spoilage (ΔE = 15.8). These results indicate that the developed films presented in this work could be effectively used as pH-monitor systems for food packaging applications, paving the way for sustainable and intelligent solutions. However, further research may be necessary to evaluate the long-term storage stability and to investigate their colorimetric response to different meat types. Extended spoilage periods, under different temperatures, should also be tested to evaluate the color response of the films to different acidic or basic volatile compounds. Additionally, further investigation is required to evaluate the biocompatibility and safety of these films for direct applications in meat packaging.

## 4. Materials and Methods

### 4.1. Chemicals and Reagents

Folin–Ciocalteu’s phenol reagent, chitosan (75–85% deacetylated, low molecular weight, 20–300 cP), D (+)-Glucose (≥99.5%), D (−)-Fructose (≥99.0%), sodium acetate trihydrate (≥99.0%), choline chloride (>98.0%), lactic acid (>98.0%), acetic acid (99.8%), 2,2- diphenyl-1-picrylhydrazyl (DPPH), and 2,2′-azino-bis (3-ethylbenzothiazoline-6-sulphonic acid) diammonium salt (ABTS) were purchased from Sigma-Aldrich (St. Louis, MO, USA). Potassium peroxydisulfate (≥99.0%) and potassium chloride (≥99.5%) were purchased from Merck KGaA (Darmstadt, Germany). Gallic acid hydrate (≥98.0%), butylene glycol (>99.0%), and 6-hydroxy-2,5,7,8-tetramethylchroman-2-carboxylic acid (Trolox) were obtained from Tokyo Chemical Industry Co., Ltd. (Tokyo, Japan). Glycerol (0.5% max water) and ethanol (99.8%) were purchased from Fisher Scientific Co. (Loughborough, UK). Sodium carbonate (≥99.8%) was purchased from Riedel de Haen (Charlotte, NC, USA). Betaine (98.0%) and Propylene glycol (99.0%) were purchased from Acros Organics (Waltham, MA, USA). Ethylene glycol (≥99.5%) was obtained from AppliChem (Darmstadt, Germany). Urea (>99.0%) was purchased from Fluka/Riedel-de Haën (Seelze, Germany). Double-distilled water (ddH_2_O) was used for all experiments. Pork meat was obtained from the local market.

### 4.2. Preparation of Natural Deep Eutectic Solvents

The preparation of NADES was performed according to the literature [81]. In brief, the hydrogen bond acceptors and the hydrogen bond donors were mixed at proper molar ratios in screw-capped glass vials and incubated at 80 °C under stirring (200 rpm) for 2 h. The formed NADES were used without further treatment, and their composition, abbreviations, and molar ratios are summarized in Table 6.

### 4.3. Wine Lees Extractions—Optimization

Red wine lees were provided from a local winery (Ioannina, Epirus, Greece), and their main characteristics are described in our previous work [2]. Before extraction, wine lees were centrifuged at 9500 rpm at 4 °C for 10 min, and the pellet was freeze-dried, ground, and stored at −20 °C until use.

The optimization of the extraction process was conducted using ethanol as a common solvent for anthocyanin extraction. The extractions were performed using an ATPIO XO-SM50 ultrasound and microwave collaborative system (Nanjing Xianou Instruments Manufacture Co., Ltd., Nanjing, China), where only the ultrasound function was used. The ultrasonic power was set at 200 watts (ultrasonic on delay: 3 s., ultrasonic turn-off time: 1 s.), and the solvent volume was 20 mL. After the extraction, the extract was centrifuged at 9500 rpm at 4 °C, for 10 min, and the supernatant was filtered through Whatman filter paper and stored at 4 °C until further use. The effect of S:s ratio, extraction time (min), and ethanol content (%) on the TPC, TAC, and AA was investigated, and the different trials are described in Table 7. After determining the optimized extraction conditions, the different NADES were tested under the same conditions as alternative solvents for ethanol.

### 4.4. Characterization of the Extracts

#### 4.4.1. Total Anthocyanin Content

The TAC of the liquid extracts was estimated using the pH-differential method, but the final volume was adjusted to 1 mL [82,83]. In brief, the dilution factor (DF) of each sample was estimated by diluting the extract with potassium chloride buffer (0.025 M, pH 1.0) to reach an absorbance in the linear range of the spectrophotometer at 520 nm. In the next step, each sample was diluted (according to the estimated DF) in potassium chloride buffer (0.025 M, pH 1.0) and sodium acetate buffer (0.4 M, pH 4.5) and left to stabilize for at least 15 min. The absorbance of both solutions was measured at 520 nm and 700 nm using a spectrophotometer (Agilent Cary 60, Santa Clara, CA, USA). The results are expressed as mg of cyanidin-3-glucoside equivalents per mL of the extract (mg C_3_GE mL^−1^) according to the following Equation (1).

TAC (mg C_3_GE mL^−1^) = A × MW × DF/ε × 1, (1)

where A = (A_520_–A_700_)_pH1.0_ − (A_520_–A_700_)_pH4.5_, and A_520_ and A_700_ are the absorbance of the solution at 520 nm and 700 nm, respectively, MW is the molecular weight of cyanidin-3-glucoside (449.2 g mol^−1^), DF is the dilution factor, and ε is the molar absorptivity (26,900 L cm^−1^ mol^−1^). The equation presented above assumes a path length of 1 cm.

#### 4.4.2. Total Phenolic Content

The TPC of the extracts was determined by the Folin–Ciocalteu assay as described in our previous work [9]. Briefly, water, a proper volume of the extract, and 10 μL of Folin–Ciocalteu reagent were mixed in a final volume of 180 μL in a 96-well plate, and the samples were incubated at room temperature for 3 min. Then, 20 μL of a 20% *w*/*v* Na_2_CO_3_ aqueous solution were added, and the samples were incubated for 1 h in the dark. The absorbance of the samples was measured at 725 nm using a UV–Vis Multiskan SkyHigh Microplate Spectrophotometer (Thermo Scientific, Waltham, MA, USA). The results are expressed in terms of gallic acid equivalents per mL of the extract (mg GAE mL^−1^) using a gallic acid standard curve.

#### 4.4.3. Antioxidant Activity DPPH—ABTS

The AA of the extracts was estimated using DPPH^•^ and ABTS^•+^ free radicals as described in our previous work [84]. In brief, a proper volume of the extracts was mixed with the DPPH^•^ and ABTS^•+^ free radicals’ solutions, and the samples were incubated in the dark for 30 min. The absorbance of the samples was measured at 517 nm and 734 nm, respectively. The AA is expressed as mg Trolox equivalents per mL of extract (mg TE mL^−1^) for both assays using Trolox standard curves.

#### 4.4.4. Color Response of the Extract in pH Changes—UV–Vis Characterization

The pH response of the final extract was evaluated by diluting the extract in buffer solutions in a pH range 2–12. The buffer solutions were prepared according to the literature [85]. In brief, the extract was diluted with each buffer (DF = 50) and left to stabilize for 15 min at room temperature. Then, 300 μL of each solution were transferred to a 96-well UV-ELISA plate, and the spectra were recorded in the 200–800 nm range using a UV–Vis Multiskan SkyHigh Microplate Spectrophotometer (Thermo Scientific, Waltham, MA, USA).

### 4.5. Preparation of pH-Responsive Chitosan Films

The preparation of chitosan films was conducted as described in our previous work with some modifications [84]. Briefly, a chitosan solution (1.5% *w*/*v*) was prepared in acetic acid (1% *v*/*v*) under stirring, at 75 °C for 30 min. After cooling, the solution was centrifuged (8000 rpm, 10 min) to remove any insoluble material. Then, 10 mL of Cs solution were mixed with 125, 250, or 500 μL (corresponding to 1.25, 2.5, or 5% *v*/*v*, respectively) of the final extract, cast in 50 mm plastic Petri dishes, and then dried at 30 °C for 24 h. Cs films without any addition and Cs films with the addition of 1.25, 2.5, or 5% *v*/*v* of extraction solvent were used as control samples.

### 4.6. Characterization of pH-Responsive Chitosan Films

#### 4.6.1. Antioxidant Activity of the Chitosan Films

The AA of the formed films was evaluated as described in our previous work with minor modifications [9]. In brief, 0.5 cm × 0.5 cm pieces of the films were mixed with the DPPH^•^ or ABTS^•+^ free radical solutions, and the absorbance of the mixtures was measured after 30 or 20 min of incubation, at 517 nm and 734 nm, respectively. The results are presented as % antioxidant activity through the following Equation (2).

AA (%) = 100 × ((A_control_ − A_sample_)/A_control_), (2)

where A_control_ and A_sample_ represent the absorbance of the free radical without and after the interaction with the extract, respectively.

#### 4.6.2. Water Swelling, and Water Solubility Assays

The water swelling (WSw), and the water solubility (WS) of the films were estimated according to the methods described in our previous work [84].

#### 4.6.3. Film Thickness

The thickness of the films was determined using a digital caliper with a resolution of 0.01 mm. At least three different measurements were performed in three different areas of the films.

#### 4.6.4. Mechanical Properties

Tensile tests were performed on strip films with a width of 10 mm and a gauge length of 30 mm using a mini tester equipped with a 1000 lb load cell. The mini tester used in this study was made by Fulam USA, while the tests were performed according to ASTM D882. Specifically, after measuring the thickness of 2 to 3 samples of each formulation with a digital micrometer, the films were clamped between the grips and tensed at a deformation speed of 10 mm min^−1^ at 25 °C. Force and deformation were recorded during the tensile experiment, and based on these data and the dimensions of the specimens (cross-sectional area and gauge length), Young’s modulus (E), tensile strength (σ_uts_), and % strain at break (ε_b_) were calculated.

#### 4.6.5. Color Response to pH Changes

To determine the pH response of the selected film, 1 cm × 1 cm pieces were immersed for 2 min in the same buffer solutions as mentioned in paragraph 4.4.4. Then, the film pieces were placed on top of a filter paper to remove the excess buffer solutions. The L (darkness/lightness), a (greenness/redness), and b (blueness/yellowness) values were determined using the Color Grab™ app (Version 3.9.2, © 2021 Loomatix Ltd., Haifa, Israel) [86], and the ΔE was calculated using the following Equation (3).

ΔE = √((L − L_0_)^2 + (a − a_0_)^2 + (b − b_0_)^2), (3)

where L_0_, a_0,_ and b_0_ are the values of the initial film, and L, a, and b are the values of the film after the interaction with the different buffer solutions. All the photos were taken in a custom-made lightbox, using a smartphone with a 48-megapixel camera. The white font of the lightbox was used as a calibration color.

#### 4.6.6. Optical Properties of the Films

The UV–Vis spectra of the films (wavelength range 200–800 nm) were obtained using a UV–Vis Multiskan SkyHigh Microplate Spectrophotometer (Thermo Scientific, Waltham, MA, USA). The film samples were cut into circular pieces and placed on a UV–ELISA plate. An empty well was used as a reference. The opacity of the films was calculated through the following equation [5,87].

Opacity (mm^−1^) = A_600_/Th, (4)

where A_600_ and Th represent the absorbance at 600 nm and the thickness (mm) of the film, respectively.

### 4.7. Monitor of Meat Freshness

The spoilage of the meat was monitored according to Chayavanich et al. [88]. In brief, 10 g of pork meat was placed in a 50 mm petri dish. One 2 cm × 2 cm piece of the film was placed on the inside of the plastic cap, and the plate was incubated at 30 °C for 24 h. Pictures of the plate were taken at 0, 1, 2, 4, and 24 h to monitor the optical color difference of the films during the spoilage.

### 4.8. Statistical Analysis

The experimental data were analyzed using one-way ANOVA analysis and Tukey’s or Dunnett’s multiple comparison tests. They were carried out using IBM SPSS Statistics version 21 (SPSS Inc., Chicago, IL, USA) to compare the mean values of each treatment and to determine the statistical significance (*p* < 0.05).

## Figures and Tables

**Table 1:** Summary of the different chitosan films cast with the addition of 50% ChCl:BG or wine lees extract in 50% ChCl:BG.

Abbreviation	Composition of Chitosan Gel (10 mL)
Cs	Chitosan 1.5%
CsNADES1	Chitosan 1.5% + 1.25% of 50% ChCl:BG 1:4
CsNADES2	Chitosan 1.5% + 2.5% of 50% ChCl:BG 1:4
CsNADES3	Chitosan 1.5% + 5% of 50% ChCl:BG 1:4
CsEx1	Chitosan 1.5% + 1.25% of wine lees extract in 50% ChCl:BG 1:4
CsEx2	Chitosan 1.5% + 2.5% of wine lees extract in 50% ChCl:BG 1:4
CsEx3	Chitosan 1.5% + 5% of wine lees extract in 50% ChCl:BG 1:4

**Table 2:** The thickness and opacity of the different chitosan films.

Film	Thickness (mm)	Opacity (mm^−1^)	Visual Characterization
Cs	0.05 ± 0.01 ^bc^	1.68 ± 0.34 ^c^	Transparent
CsNADES1	0.05 ± 0.00 ^bc^	1.70 ± 0.00 ^c^	Transparent
CsNADES2	0.06 ± 0.00 ^bc^	1.58 ± 0.00 ^c^	Transparent
CsNADES3	0.08 ± 0.01 ^a^	1.63 ± 0.11 ^c^	Transparent
CsEx1	0.05 ± 0.01 ^c^	3.73 ± 0.81 ^b^	Translucent
CsEx2	0.06 ± 0.01 ^bc^	4.89 ± 0.83 ^b^	Translucent
CsEx3	0.07 ± 0.00 ^ab^	6.24 ± 0.00 ^a^	Translucent/Opaque

Different lowercase letters indicate differences in significance (*p* ≤ 0.05) in Tukey’s multiple range test.

**Table 3:** The WSw and WS properties of the films.

Film	WSw (%)	WS (%)
Cs	2038.9 ± 337.8 ^a^	41.5 ± 2.1 ^b^
CsNADES1	133.4 ± 13.6 ^b^	19.7 ± 0.8 ^d^
CsNADES2	75.4 ± 47.3 ^b^	31.4 ± 0.3 ^c^
CsNADES3	0.0 ± 0.0 ^b^	47.7 ± 0.3 ^a^
CsEx1	67.6 ± 33.5 ^b^	20.9 ± 1.7 ^d^
CsEx2	96.5 ± 19.3 ^b^	26.7 ± 1.2 ^c^
CsEx3	0.0 ± 0.0 ^b^	39.1 ± 0.7 ^b^

Different lowercase letters indicate differences in significance (*p* ≤ 0.05) in Tukey’s multiple range test.

**Table 4:** Antioxidant activity of the different chitosan films.

	% Antioxidant Activity	
Film	ABTS	DPPH
Cs	32.0 ± 6.4 ^de^	10.1 ± 4.8 ^c^
CsNADES1	39.1 ± 0.3 ^cd^	7.4 ± 0.3 ^c^
CsNADES2	23.3 ± 0.8 ^ef^	8.1 ± 1.3 ^c^
CsNADES3	16.5 ± 2.7 ^f^	11.2 ± 1.9 ^c^
CsEx1	49.2 ± 0.2 ^c^	11.0 ± 2.9 ^c^
CsEx2	74.1 ± 3.2 ^b^	30.3 ± 3.5 ^b^
CsEx3	91.6 ± 0.8 ^a^	61.2 ± 2.9 ^a^

Different lowercase letters indicate differences in significance (*p* ≤ 0.05) in Tukey’s multiple range test.

**Table 5:** L, a, b and ΔE values of the CsEx2 film after the immersion in the different pH values.

	L	a	b	ΔΕ
Initial Film	51.0 ± 0.2 ^a^	−5.6 ± 0.1 ^g^	4.9 ± 0.0 ^cde^	-
pH 2.0	43.3 ± 0.7 ^bc^	26.7 ± 0.5 ^a^	7.8 ± 0.6 ^a^	33.3 ± 0.6 ^a^
pH 3.0	38.0 ± 1.7 ^e^	22.7 ± 0.1 ^b^	4.5 ± 0.1 ^cde^	31.2 ± 0.8 ^a^
pH 4.0	39.4 ± 0.3 ^cde^	16.6 ± 0.0 ^c^	7.4 ± 0.0 ^ab^	25.2 ± 0.1 ^b^
pH 5.0	49.9 ± 1.3 ^a^	4.2 ± 0.6 ^d^	6.5 ± 1.1 ^abc^	10.1 ± 0.6 ^ef^
pH 6.0	38.9 ± 0.1 ^de^	0.7 ± 0.4 ^e^	3.8 ± 0.3 ^de^	13.7 ± 0.1 ^d^
pH 7.0	42.7 ± 0.5 ^bcd^	−3.6 ± 0.4 ^f^	7.7 ± 1.1 ^a^	9.0 ± 0.9 ^ef^
pH 8.0	44.7 ± 0.5 ^b^	−3.2 ± 0.3 ^f^	4.6 ± 0.1 ^cde^	6.8 ± 0.6 ^f^
pH 9.0	32.1 ± 2.4 ^f^	−3.4 ± 0.1 ^f^	2.9 ± 0.1 ^ef^	19.1 ± 2.4 ^c^
pH 10.0	38.9 ± 0.1 ^de^	−7.9 ± 0.4 ^h^	5.4 ± 0.3 ^bcd^	12.4 ± 0.2 ^ed^
pH 11.0	44.7 ± 0.2 ^b^	−14.4 ± 0.3 ^j^	1.4 ± 0.5 ^f^	11.4 ± 0.3 ^ed^
pH 12.0	33.0 ± 0.6 ^f^	−12.1 ± 0.2 ^i^	1.2 ± 0.4 ^f^	19.5 ± 0.5 ^c^

Different lowercase letters indicate differences in significance (*p* ≤ 0.05) in Tukey’s multiple range test.

**Table 6:** The composition, molar ratio, and abbreviations of the different NADES.

Abbreviation	Hydrogen Bond Acceptor	Hydrogen Bond Donor	Molar Ratio
Bet:BG (1:4)	Betaine	Butylene glycol	1:4
Bet:EG (1:3)	Betaine	Ethylene glycol	1:3
Bet:Gly (1:3)	Betaine	Glycerol	1:3
Bet:LaA (1:2)	Betaine	Lactic acid	1:2
Bet:PG (1:4)	Betaine	Propylene glycol	1:4
ChCl:BG (1:4)	Choline Chloride	Butylene glycol	1:4
ChCl:EG (1:2)	Choline Chloride	Ethylene glycol	1:2
ChCl:Gly(1:2)	Choline Chloride	Glycerol	1:2
ChCl:LaA (1:2)	Choline Chloride	Lactic acid	1:2
ChCl:PG (1:3)	Choline Chloride	Propylene glycol	1:3
ChCl:U (1:2)	Choline Chloride	Urea	1:2
ChCl:Fru:H_2_O (5:2:5)	Choline Chloride	Fructose, H_2_O	5:2:5
ChCl:Glc:H_2_O (5:2:5)	Choline Chloride	Glucose, H_2_O	5:2:5
ChCl:Gly:EG (1:1:1)	Choline Chloride	Glycerol, Ethylene glycol	1:1:1
ChCl:U:EG (1:1:1)	Choline Chloride	Urea, Ethylene glycol	1:1:1
ChCl:U:Gly (1:1:1)	Choline Chloride	Urea, Glycerol	1:1:1

**Table 7:** Extraction optimization tested conditions.

Optimization Parameter	Tested Conditions
Solid to solvent ratio (S:s, g mL^−1^)	1:5, 1:10, 1:20, 1:30, 1:40, 1:50
Extraction time (min)	5, 10, 15, 20, 25, 30
Ethanol content (%)	0, 25, 50, 75, 100

## Data Availability

The data are contained within the article.

## Abbreviations

The following abbreviations are used in this manuscript:

TAC	Total anthocyanin content
TPC	Total phenolic content
AA	Antioxidant activity
ABTS	2,2′-azino-bis (3-ethylbenzothiazoline-6-sulphonic acid) diammonium salt
DPPH	2,2- diphenyl-1-picrylhydrazyl
NADES	Natural Deep Eutectic Solvents
UAE	Ultrasound-assisted extraction
GAE	Gallic acid equivalents
C_3_GE	Cyanidin-3-glucoside equivalents
TE	Trolox equivalents
Cs	Chitosan
Ch:Cl	Choline Chloride
Bet	Betaine
BG	Butylene glycol
Gly	Glycerol
PG	Propylene glycol
EG	Ethylene glycol
LaA	Lactic acid
U	Urea
Glc	Glucose
Fru	Fructose
DF	Dilution factor
MW	Molecular weight
E	Young’s modulus
σuts	Tensile strength
εb	Strain at break
WSw	Water swelling
WS	Water solubility
CsNADES1	Chitosan film containing 1.25% of 50% ChCl:BG 1:4
CsNADES2	Chitosan film containing 2.5% of 50% ChCl:BG 1:4
CsNADES3	Chitosan film containing 5% of 50% ChCl:BG 1:4
CsEX1	Chitosan film containing 1.25% of extract in 50% ChCl:BG 1:4
CsEX2	Chitosan film containing 2.5% of extract in 50% ChCl:BG 1:4
CsEX3	Chitosan film containing 5% of extract in 50% ChCl:BG 1:4
